# Characteristics and Outcomes of New-Onset Cardiomyopathy in Hospitalized COVID-19 Patients

**DOI:** 10.3390/jcm14093258

**Published:** 2025-05-07

**Authors:** Sachin Kumar, Gautam Shah, Raunak Nair, Sarah Rikabi, Mohannad Seif, Bindesh Ghimire, Brian Griffin, Umesh N. Khot

**Affiliations:** 1 Department of Cardiovascular Medicine, Mount Sinai Morningside, New York, NY 10025, USA; 2 Department of Cardiovascular Medicine, Heart, Vascular, and Thoracic Institute, Cleveland Clinic, Cleveland, OH 44195, USA; 3 Department of Internal Medicine, Cleveland Clinic Fairview Hospital, Cleveland, OH 44111, USA

**Keywords:** COVID-19, cardiomyopathy, echocardiography, left ventricular dysfunction

## Abstract

**Background:** The association between Coronavirus Disease-2019 (COVID-19) and new-onset cardiomyopathy (NOC) is unclear. **Objectives:** We aim to assess the incidence of NOC in hospitalized COVID-19 patients and its impact on short- and long-term survival. **Methods:** We retrospectively studied 2219 COVID-19 patients hospitalized between March 2020 and February 2022 who underwent an in-hospital echocardiogram. NOC was defined as a left-ventricular ejection fraction (LVEF) reduction of >10%, resulting in an LVEF of <54% for females and <52% for males. The 30-day and 1-year survival outcomes in patients without and with NOC were studied. **Results:** Among 25,943 hospitalized COVID-19 patients, 2219 met our inclusion criteria, with 209 (9.4%) having NOC. NOC patients were more likely to be male (56.1% vs. 68.4%, *p* = 0.001) and have chronic kidney disease (51.4% vs. 60.3%, *p* = 0.018). They had a higher 30-day mortality rate (29.1% vs. 32%, *p* = 0.033), but the 1-year survival rate was similar between the patients without and with NOC (36.9% vs. 41.6%, *p* = 0.12). Multivariable regression revealed that advanced age, admission to intensive care unit, mechanical ventilation, treatment with glucocorticoids, and treatment with vasopressors were associated with higher odds of 30-day mortality in NOC patients. Only 74 (35.4%) NOC patients had follow-up echocardiograms after discharge, of which 47 showed persistent cardiomyopathy. **Conclusions:** NOC can affect around 1 out of 10 hospitalized COVID-19 patients undergoing echocardiography. While NOC was associated with worse short-term survival, it did not impact the long-term mortality of these patients. Persistent LVEF deficits in some patients emphasize the need for improved outpatient follow-up to identify at-risk individuals and optimize treatment.

## 1. Introduction

The Coronavirus Disease-2019 (COVID-19) pandemic caused by severe acute respiratory syndrome Coronavirus-2 (SARS-CoV-2) led to an unprecedented global health crisis with over 777 million cases and more than 7 million deaths worldwide as of March 2025 [1]. While COVID-19 primarily affects the respiratory system, there is robust evidence confirming the detrimental effects of this virus on the cardiovascular system, including but not limited to hypercoagulability, myocarditis, pericarditis, cardiomyopathy, and arrhythmias [2,3]. Among the cardiovascular complications of COVID-19, myocardial injury is among the most common, with up to 30% of hospitalized COVID-19 patients exhibiting elevated troponin levels [4,5,6].

SARS-CoV-2 infection can precipitate new-onset cardiomyopathy through several interrelated pathophysiologic mechanisms. Direct myocardial damage may arise from the viral invasion of cardiomyocytes via angiotensin converting enzyme 2 (ACE2) receptors in cardiac tissue, potentially resulting in viral myocarditis or direct cytopathic injury [7]. Concurrently, severe COVID-19 often evokes a pronounced immune response (cytokine storm), with markedly elevated levels of proinflammatory cytokines that can suppress cardiac contractility and injure the myocardium in the absence of direct viral infiltration [8]. Endothelial cell infection and inflammation further foster a hypercoagulable state, promoting microvascular thrombosis and compromised myocardial perfusion [9]. Additionally, the excessive sympathetic stimulation and dysregulation of the renin–angiotensin–aldosterone system, secondary to ACE2 downregulation, can exacerbate left ventricular dysfunction [7]. These processes are validated by cardiac MRI findings in COVID-19 patients, which frequently demonstrate myocardial edema, inflammation, and fibrosis, even among individuals without prior cardiac disease [10,11]. Large clinical cohorts similarly note elevated rates of incident heart failure, myocarditis, and other cardiomyopathies in the post-COVID-19 setting, underscoring the important clinical implications of these mechanistic insights [12].

Numerous studies have investigated echocardiographic findings in patients with COVID-19 infection. However, they have primarily focused on right ventricular function in COVID-19 patients, and less is known about the effect of this disease on left ventricular function [13,14,15,16,17,18]. Furthermore, it is unclear whether the reported left ventricular dysfunction in acute COVID-19 infection is new or preexisting, as most studies lack comparison between echocardiograms performed during the acute phase of illness and baseline echocardiograms performed before the infection, limiting their ability to identify true new-onset cardiomyopathy (NOC) [19,20,21]. Our study uniquely addresses this gap by leveraging pre- and in-hospital echocardiograms to identify truly new left ventricular systolic dysfunction and assess its clinical relevance. The present study aims to evaluate the characteristics and outcomes of new-onset left ventricular systolic dysfunction observed in hospitalized COVID-19 patients. In doing so, we hope to study the incidence and effect of this newly defined pathology on clinical outcomes in this known cohort of patients.

## 2. Materials and Method

We conducted a retrospective cohort study of COVID-19 patients hospitalized at ten Cleveland Clinic hospitals between 18 March 2020 and 25 February 2022. Confirmation of COVID-19 was based on a positive reverse transcription-polymerase chain reaction assay or another validated laboratory test (e.g., rapid molecular assay) on nasopharyngeal or oropharyngeal swabs. The study included patients who had received an in-hospital echocardiogram for any clinical reason and had a prior echocardiogram available for comparison. We defined NOC as a reduction in the left-ventricular ejection fraction (LVEF) of >10% compared to a prior echocardiogram, with a new LVEF of <54% in females and <52% in males. The specific LVEF cutoff values were chosen based on the American Society of Echocardiography Recommendations for Cardiac Chamber Quantification by Echocardiography in Adults [22]. All echocardiograms were performed by certified cardiac sonographers using standardized protocols, and studies were interpreted by board-certified cardiologists. The primary measure of left ventricular function was the LVEF. LVEF was determined using the visual estimate or the biplane method of disks (modified Simpson’s rule). The study received proper ethical oversight and was approved by the Cleveland Clinic Institutional Review Board (IRB#: 20480). Relevant laboratory parameters at admission and during hospitalization (e.g., white blood cell count, inflammatory markers, BNP/NT-proBNP levels, cardiac troponins, and others) were recorded when available. Data regarding comorbidities (hypertension, diabetes mellitus, coronary artery disease, heart failure, chronic kidney disease), medication history, and inpatient treatment (e.g., corticosteroids, vasopressors, mechanical ventilation) were extracted from the electronic medical record. We also noted each patient’s hemodynamic status and need for ICU-level care.

Descriptive statistics were utilized to summarize the data obtained. Continuous variables were presented as the median [Quartile 1 and Quartile 3] and were compared using the Wilcoxon rank-sum test, analysis of variance, or the Kruskal–Wallis test (for ≥2 groups). Categorical data were described using frequencies and percentages and were compared using the chi-squared test. Multivariable logistic regression was performed to analyze the impact of clinically significant covariates on 30-day outcomes in patients with NOC. All variables were initially assessed in univariable linear regression analysis; those with a *p*-value < 0.05 and clinically important variables were included in multivariable linear regression analysis. We then used backward stepwise regression with final model selection based on the lowest Akaike Information Criterion. Collinearity was assessed with variance inflation factors. Finally, survival analysis was performed using the Kaplan–Meier nonparametric method, and comparisons were made using the log-rank test. The primary outcome of interest was 30-day all-cause mortality, and the secondary outcome of interest was 1-year all-cause mortality.

## 3. Results

**Figure 1:**
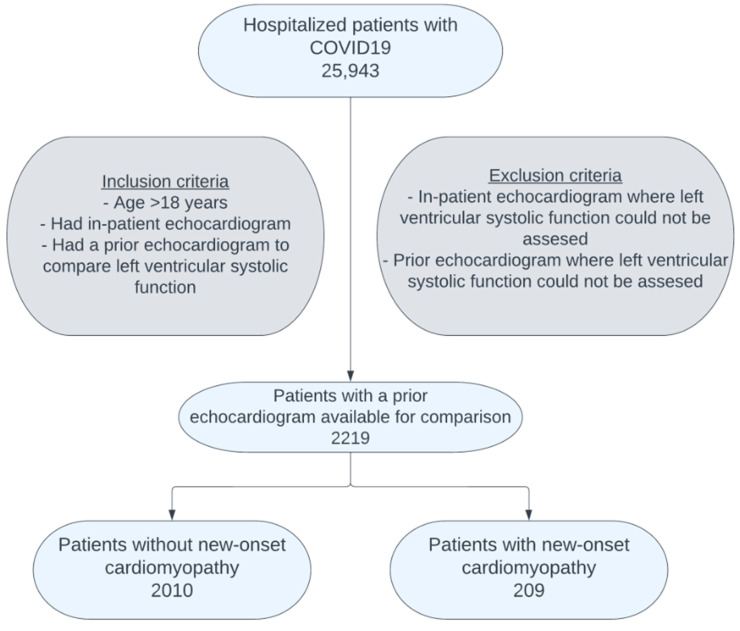
Patient selection: Out of the 25,943 hospitalized patients with COVID-19, 2219 had an inpatient echocardiogram that could be compared with an echocardiogram performed before admission. Of these, 209 were found to have new-onset cardiomyopathy.

Among the 25,943 hospitalized COVID-19 patients in the Cleveland Clinic registry, 3829 underwent an in-hospital echocardiogram. Of these, 2219 patients had a prior echocardiogram for comparison (Figure 1). The mean time interval between the pre-COVID-19 hospitalization echocardiogram and in-hospital echocardiogram was 2.9 ± 3.2 years.

Out of our final cohort of 2219 patients, 209 (9.4%) developed NOC. Patients without NOC had a mean baseline LVEF of 58% ± 10%, which remained relatively unchanged at 58% ± 11% during hospitalization. In contrast, patients in the NOC group had a mean baseline LVEF of 55% ± 12%, which declined to 45% ± 12% during their hospital stay, which is consistent with the defined criteria for NOC. The comparison of baseline characteristics revealed that, compared to patients without NOC, patients with NOC were more likely to be male (56.1% vs. 68.4%, *p* = 0.001) and have preexisting chronic kidney disease (51.4% vs. 60.3%, *p* = 0.018). Elevated high-sensitivity troponin, available for only 907 patients, was associated with the increased risk of developing NOC (OR-2.014, 95% CI 1.219–3.363, *p* = 0.007). There was no significant difference in the prevalence of smoking (51.6% vs. 53.1%, *p* = 0.730), coronary artery disease (36.6% vs. 39.7%, *p* = 0.411), or heart failure (40.9% vs. 46.9%, *p* = 0.113) between the patients without and with NOC (Table 1). Patients with NOC were more likely to be on beta-blockers (BBs) (68.7% vs. 77%, *p* = 0.015) and angiotensin-converting enzyme inhibitors or angiotensin receptor blockers (ACEi/ARBs) (52.3% vs. 62.2%, *p* = 0.008).

**Figure 2:**
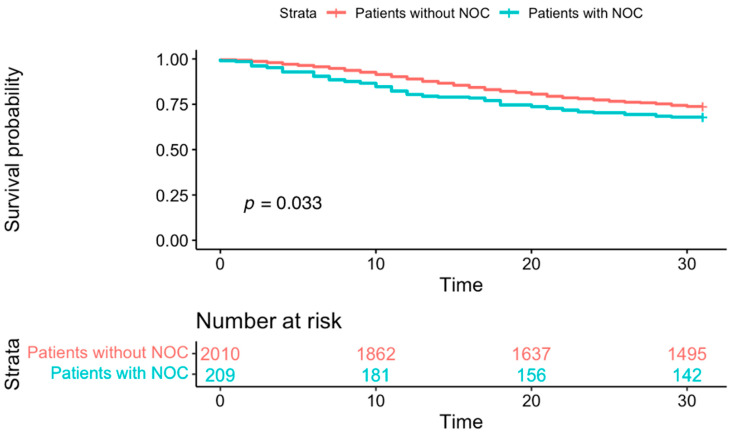
Comparison of 30-day mortality between patients without and with NOC: Kaplan–Meier curve showing that relative to patients without NOC, those with NOC had a higher 30-day all-cause mortality (29.1% vs. 32%, *p* = 0.033).

**Figure 3:**
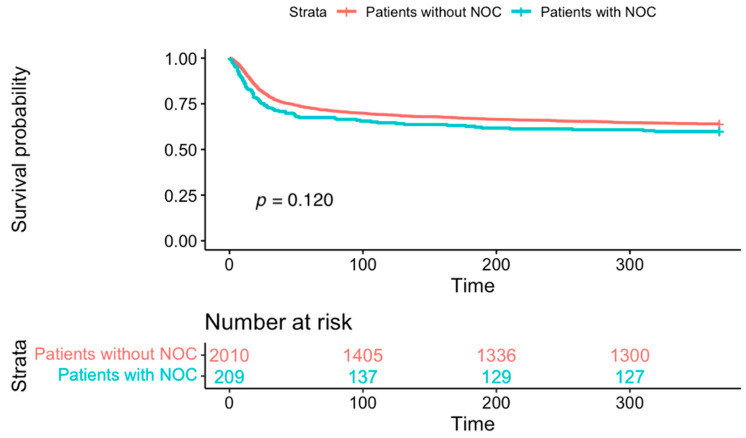
Comparison of 1-year mortality between patients without and with NOC: Kaplan–Meier curve showing no difference in the 1-year all-cause mortality between patients without and with NOC (36.9% vs. 41.6%, *p* = 0.12).

Relative to patients without NOC, those with NOC had a higher unadjusted 30-day all-cause mortality (29.1% vs. 32%, *p* = 0.033) (Figure 2). There was no difference in the 1-year all-cause mortality between patients without NOC and with NOC (36.9% vs. 41.6%, *p* = 0.12) (Figure 3). In patients with NOC, multivariable regression analysis adjusting for baseline factors showed that factors including older age [OR: 1.03 (97.5% CI: 1.02–1.04), *p* < 0.001)], ICU admission [OR: 1.96 (97.5% CI: 1.44–2.65), *p* < 0.001], mechanical ventilation [OR: 2.62 (1.85–3.71), *p* < 0.001], glucocorticoid treatment [OR: 1.99 (97.5% CI: 1.48–2.69), *p* < 0.001], and vasopressor use [OR: 3.40 (95% CI: 2.43–4.74), *p* < 0.001] were independently associated with higher odds of 30-day mortality (Table 2).

**Figure 4:**
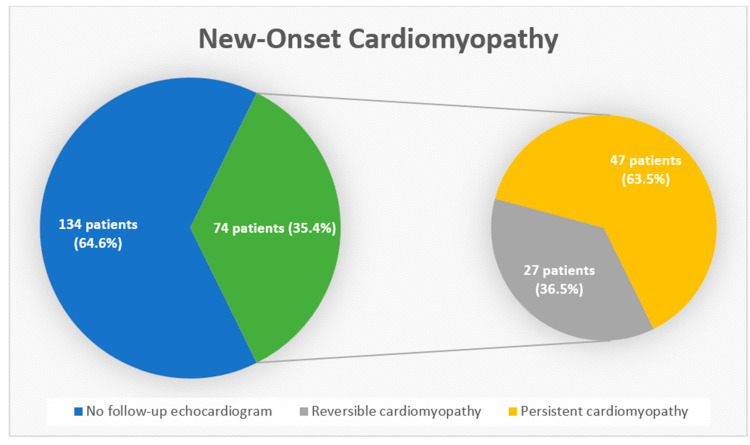
Follow-up echocardiograms shown in pie charts: Of the patients with NOC, 64.6% did not receive a follow-up echocardiogram (blue) after their discharge. The majority of patients who received a follow-up echocardiogram (green) were found to have persistent cardiomyopathy.

Among the 209 patients with NOC, only 74 (35.4%) received a follow-up echocardiogram. Of these, 47 patients had persistent cardiomyopathy (Figure 4), defined as an LVEF of <54% in females and <52% in males. The mean time interval between the in-hospital and post-hospitalization echocardiograms in patients with NOC was 129 ± 97 days.

## 4. Discussion

Our study represents the most extensive investigation to date assessing left ventricular dysfunction in hospitalized COVID-19 patients via echocardiography that uniquely compares in-hospital echocardiograms with those performed before the patients’ COVID-19 diagnosis. Our study introduces a distinct cohort of COVID-19 patients with NOC, which accounted for a noteworthy 10% of the hospitalized patients undergoing echocardiography. We found that relative to the patients without NOC, those with NOC had a significantly higher 30-day mortality rate; however, the two groups had similar 1-year mortality. Advanced age and critical illness, rather than NOC, were responsible for the higher 30-day mortality in these patients. Despite the high prevalence of NOC, only a third of these patients underwent follow-up echocardiograms. This is particularly concerning given that a persistent LVEF dysfunction was found in over half of the patients with NOC who had follow-up echocardiography. These findings emphasize the need for enhanced outpatient monitoring, thereby potentially improving outcomes in heart failure management.

Our study revealed that among the COVID-19 patients undergoing echocardiography for any reason, approximately one in ten patients experienced a reduction in LVEF. Multiple mechanisms of such cardiac involvement in COVID-19 have been proposed, which include direct injury by SARS-CoV-2 invading the cardiac myocytes and indirect injury caused by hypoxia, the marked elevation of inflammatory mediators, catecholamines, and pro-thrombotic agents [23,24,25,26,27,28,29]. Our findings are consistent with prior studies that examined left ventricular systolic dysfunction in hospitalized COVID-19 patients, although reported incidence has varied depending on the population studied and imaging protocols used [15,18,30]. While early observational data from Wuhan suggested that approximately one-quarter of patients developed acute heart failure, systematic echocardiographic studies have generally reported lower rates of new-onset LV dysfunction ranging from 10 to 27% [31]. For example, Szekely et al. identified reduced LVEF < 50% in 10% of patients upon admission, whereas van den Heuvel et al. reported new LV impairment in 27% of patients, defined by an abnormal strain or EF < 52% [32]. Similarly, a multinational ICU-based echocardiographic study found that approximately one-third of critically ill COVID-19 patients had evidence of ventricular dysfunction, including both left- and right-sided involvement [18,33]. These prevalence estimates closely parallel our observed incidence of NOC (9.4%) in patients with baseline echocardiographic data, supporting the external validity of our findings. Uniquely, by comparing the echocardiograms obtained before and after the diagnosis of COVID-19, our study captures the dynamic evolution of cardiac function observed in patients during their COVID-19 hospitalization. Patients with NOC were more likely to be on BBs and ACEi/ARBs. We hypothesize that this observation was not due to the difference in the prevalence of comorbidities such as coronary artery disease, congestive heart failure, or hypertension in the two cohorts. This is supported by the equal distribution of these comorbidities among the cohorts of patients without and with NOC.

In addition to incidence, several studies have underscored the prognostic implications of ventricular dysfunction in COVID-19. Echocardiographic markers such as reduced LVEF, RV dilation, or impaired TAPSE have been independently associated with worse outcomes, including ICU admission and in-hospital mortality [19,34]. We found that in comparison to patients without NOC, those with NOC had a higher 30-day mortality. This finding is consistent with previous studies reporting an association between cardiac dysfunction and poor short-term outcomes in COVID-19 patients [16,35,36,37,38]. However, our study went beyond these prior findings by conducting a multivariable analysis to uncover the true nature of this association. Remarkably, we found that the NOC itself was not responsible for the increased mortality rate, but rather, it was other predictors of disease severity such as advanced age, admission to intensive care, mechanical ventilation, treatment with glucocorticoids, and treatment with vasopressors that were responsible for the higher 30-day mortality in patients with NOC. This novel finding suggests that the link between NOC and mortality is not causative but rather multifactorial and associative. Our findings further imply that NOC serves as a marker of severity in the spectrum of acute COVID-19 illness. Since the presence of NOC is associated with poorer outcomes, it is crucial to identify such patients early and initiate aggressive treatment strategies to optimize their chances of recovery and minimize adverse consequences. Importantly, our study also revealed that the 1-year survival rate in both groups was similar after recovery from acute COVID-19 infection. This suggests that the impact of NOC on the outcomes of COVID-19 patients is a transient phenomenon related to the acute phase of the disease rather than a chronic condition with long-term consequences. It is important to acknowledge that our study establishes the association and describes the interplay between NOC and illness severity in patients hospitalized with COVID-19 infection. It does not establish a causative association between these two entities. Furthermore, the utilization of coronary angiography and cardiac magnetic resonance imaging (MRI) in these real-world cohorts of patients was significantly limited for further evaluation of the cause of underlying myocardial injury in these patients. Consequently, differentiating alternative causes of cardiomyopathy, such as ischemic cardiomyopathy, preexisting non-ischemic dilated cardiomyopathies (e.g., from viral myocarditis or familial causes), stress-induced (Takotsubo) cardiomyopathy, tachycardia-induced cardiomyopathy, hypertrophic cardiomyopathy, infiltrative processes (such as cardiac amyloidosis or sarcoidosis), and significant valvular heart disease must be considered when evaluating any new reduction in LVEF.

There are very limited data available on the chronic course and progression of decreased LVEF during acute COVID-19 illnesses [39]. The study by Salah, H. et al. highlights the increased incidence of heart failure in post-recovery COVID-19 patients, identifying the association between COVID-19 hospitalization and the increased risk of incident heart failure [40]. However, other contemporary cohorts have shown that myocardial dysfunction during COVID-19 is often transient, with follow-up echocardiography demonstrating the normalization of LV function in the majority of survivors [41,42]. In our study, upon the follow-up of patients with NOC, we found that only one-third of these patients received a repeat echocardiogram after discharge. Interestingly, over half of the patients who underwent repeat echocardiography continued to have persistent deficits in their LVEF. The low rate of follow-up echocardiograms among patients with NOC indicates a missed opportunity for the early detection and management of persistent LV dysfunction and highlights the importance of improved outpatient follow-up. Such monitoring would facilitate the early identification of patients requiring additional intervention for their heart failure management, thus potentially improving their outcomes. Prospective studies incorporating cardiac MRI and detailed phenotyping are critical in distinguishing transient versus sustained myocardial injury and understanding the pathophysiology driving these changes. Additionally, developing risk stratification tools that combine echocardiographic findings, clinical severity, and laboratory data may help identify patients at risk of developing NOC so we can target interventions in those most likely to benefit from early treatment. As viral strains and vaccination coverage continue to evolve, understanding their influence on cardiac sequelae will be essential to optimizing care in future waves of COVID-19 and related respiratory illnesses.

## 5. Limitations

Our study has certain limitations. Since the data collection was retrospective and via manual health record extraction, it is subject to reporting and ascertainment bias. Although our study represents the most extensive comparison of pre- and post-diagnosis echocardiograms to date to evaluate the impact of COVID-19 on left ventricular function, given the global scale of the COVID-19 pandemic, our sample size is relatively small. Additionally, cardiac biomarker data were not consistently available for all patients and, hence, are not included in our final analysis. The lack of coronary angiography and cardiac MRI data limits our ability to characterize the underlying myocardial pathology contributing to NOC. There were limited data on the use of other guidelines directed at cardiac medications, which could have influenced cardiac function and outcomes. Furthermore, among patients with NOC, follow-up echocardiography was more likely obtained in those with persistent symptoms or clinical concerns, potentially overestimating the true prevalence of sustained left ventricular dysfunction in the overall cohort. Our study is also subject to substantial case selection bias, as only hospitalized patients with clear clinical indications or at higher risk on clinical grounds underwent echocardiography. Thus, our study population may not fully represent all individuals with COVID-19. Lastly, we did not have the exact data on the specific SARS-CoV-2 variants in our patients or their vaccination status. The common infecting strains during the study period were alpha, delta, and the early part of the omicron variant.

## 6. Conclusions

In this large, multicenter cohort of hospitalized COVID-19 patients, NOC occurred in nearly 10% of those undergoing echocardiography and was associated with higher short-term mortality, largely driven by the severity of illness. Over half of patients with NOC who underwent follow-up imaging had persistent LV dysfunction. These findings highlight the need to identify patients at higher risk of developing NOC during hospitalization and to ensure structured outpatient follow-up, both of which are critical for early heart failure intervention and improved long-term outcomes.

## Figures and Tables

**Table 1:** Baseline characteristics of patients without and with new-onset cardiomyopathy.

Characteristics		Patients Without New-Onset Cardiomyopathy 2010	Patients with New-Onset Cardiomyopathy 209	*p* Value
Patient Characteristics	Age (median [IQR])	69.72 [60.30, 78.65]	70.83 [61.83, 78.22]	0.423
	Male sex	1127 (56.1%)	143 (68.4%)	0.001
	Smoking	1037 (51.6%)	111 (53.1%)	0.730
	BMI (median [IQR])	28.82 [24.70, 34.54]	27.87 [24.00, 34.15]	0.294
	COPD	457 (22.7%)	47 (22.5%)	1.000
	Malignancy	450 (22.4%)	44 (21.1%)	0.723
	Diabetes	842 (41.9)	93 (44.5%)	0.514
	Hypertension	1516 (75.4%)	149 (71.3%)	0.219
	Coronary artery disease	735 (36.6%)	83 (39.7%)	0.411
	Heart failure	823 (40.9%)	98 (46.9%)	0.113
	Pre-hospitalization EF (mean ± SD)	58% ± 10%	55% ± 12%	>0.05
	Atrial fibrillation	937 (46.6%)	110 (52.6%)	0.113
	Chronic kidney disease	1034 (51.4%)	126 (60.3%)	0.018
	End-stage renal disease	233 (11.6%)	23 (11.0%)	0.889
	Obstructive sleep apnea	730 (36.3%)	79 (37.8%)	0.728
	ACEi/ARBs	1051 (52.3%)	130 (62.2%)	0.008
	Beta-blockers	1380 (68.7%)	161 (77%)	0.015
	Statin	1333 (66.3%)	151 (72.2%)	0.098
	Aspirin	1200 (59.7%)	136 (65.1%)	0.151
In-Hospital Treatment	Supplemental Oxygen	1559 (77.6%)	161 (77.0%)	0.931
	Glucocorticoids	1363 (67.8%)	134 (64.1%)	0.314
	Convalescent plasma	51 (2.5%)	6 (2.9%)	0.952
	Therapeutic anticoagulation	1052 (52.3%)	126 (60.3%)	0.034
	Vasopressors	674 (33.5%)	79 (37.8%)	0.245
	ACEi/ARBs	589 (29.3%)	85 (40.7%)	0.001
	Beta-blockers	1325 (65.9%)	169 (80.9%)	<0.001
Inpatient Characteristics	Hospital length of stay (median [IQR])	11.00 [6.00, 20.00]	10.00 [5.00, 18.00]	0.040
	Use of non-invasive ventilation	819 (40.7%)	90 (43.1%)	0.566
	Mechanical ventilation	585 (29.1%)	67 (32.1%)	0.417
	Admission to ICU	1132 (56.3%)	123 (58.9%)	0.529
	ICU length of stay (median [IQR])	5.00 [3.00, 12.00]	5.00 [3.00, 11.00]	0.298

BMI, body metabolic index; IQR, interquartile range; COPD, chronic obstructive pulmonary disease; SD, standard deviation; ACEi, angiotensin-converting enzyme inihibitors; ARBs, angiotensin receptor blockers; ICU, intensive care unit.

**Table 2:** Factors associated with 30-day mortality in patients with NOC.

Variable	Univariate Analysis OR (95% CI)	*p* Value	Multivariable Analysis OR (97.5% CI)	*p* Value
Age	1.02 (1.01–1.02)	<0.001	1.03 (1.02–1.04)	<0.001
Female	0.96 (0.79–1.16)	<0.001	0.94 (0.75–1.18)	0.58
BMI	0.99 (0.98–1.00)	<0.001	0.99 (0.98–1.01)	0.34
COPD	1.34 (1.07–1.66)	<0.001	1.17 (0.90–1.51)	0.24
Chronic kidney disease	1.24 (1.02–1.50)	<0.001	1.11 (0.89–1.39)	0.35
ACEi/ARBs during admission	0.60 (0.48–0.75)	<0.001	0.83 (0.65–1.07)	0.15
Beta-blockers during admission	0.69 (0.57–0.84)	<0.001	0.80 (0.61–1.00)	0.05
New-onset cardiomyopathy	1.32 (0.97–1.79)	<0.001	1.13 (0.78–1.65)	0.51
Glucocorticoid treatment	3.01 (2.39–3.84)	<0.001	1.99 (1.48–2.69)	<0.001
Hospital length of stay	0.98 (0.97–0.99)	<0.001	0.92 (0.91–0.93)	<0.001
Supplemental oxygen	3.15 (2.39–4.23)	<0.001	1.26 (0.87–1.82)	0.23
Mechanical ventilation	4.18 (3.42–5.11)	<0.001	2.62 (1.85–3.71)	<0.001
Admission to ICU	3.62 (2.93–4.51)	<0.001	1.96 (1.44–2.65)	<0.001
Vasopressor requirement	4.77 (3.91–5.83)	<0.001	3.40 (2.43–4.74)	<0.001

NOC, new-onset cardiomyopathy; OR, odds ratio; CI, confidence interval; BMI, body mass index; COPD, chronic obstructive pulmonary disease; ACEi, angiotensin-converting enzyme inhibitors; ARBs, angiotensin receptor blockers; ICU, intensive care unit.

## Data Availability

The datasets presented in this article are patient specific and hence not readily available. Requests to access the datasets should be directed to Gautam Shah.
